# Supplementary material for: HLA-class II genes association with multiple sclerosis: An immunogenetic prediction among multiple sclerosis Jordanian patients
Source: PLoS One. 2025 Feb 25;20(2):e0318824. doi: 10.1371/journal.pone.0318824 (PMC11856260; doi:10.1371/journal.pone.0318824)
Supplement: S1 Table — N: number of volunteers, Pc: Corrected P value ≤ 0.013 OR: odds ratio, CI: Confidence Interval. (DOCX) [file pone.0318824.s001.docx]

**HLA-Class II Genes Association with Multiple Sclerosis: An Immunogenetic Prediction Among Multiple Sclerosis Jordanian Patients**

Sawsan I. Khdair^1,*^, Lubna Al-Khareisha^1,2^, Osama H. Abusara^1^, Alaa M. Hammad^1^, Alaa Khudair^3^

^1^ Faculty of Pharmacy, Al-Zaytoonah University of Jordan, Amman 11733, Jordan

^2^ Department of Pharmacy, Al-Bashir Hospital, Amman, Jordan

^3^ Faculty of Dentistry, Jordan University of Science and Technology, Amman, Jordan

^*^ Corresponding author:

E-mail: sawsan.khdair@zuj.edu.jo (S.I. Khdair).

**Sawsan I. Khdair**: Faculty of Pharmacy, Al-Zaytoonah University of Jordan, Amman 11733, Jordan; [sawsan.khdair@zuj.edu.jo](mailto:sawsan.khdair@zuj.edu.jo); <https://orcid.org/0000-0002-1555-1062>

**Lubna Al-Khareisha**: Department of Pharmacy, Al-Bashir Hospital, Amman, Jordan; Faculty of Pharmacy, Al-Zaytoonah University of Jordan, Amman 11733, Jordan; alkhreishahlubna@gmail.com; https://orcid.org/0009-0001-6662-5949

**Osama H. Abusara**: Faculty of Pharmacy, Al-Zaytoonah University of Jordan, Amman 11733, Jordan; [o.abusara@zuj.edu.jo](mailto:o.abusara@zuj.edu.jo); <https://orcid.org/0000-0002-0856-5618>

**Alaa M. Hammad**: Faculty of Pharmacy, Al-Zaytoonah University of Jordan, Amman 11733, Jordan; alaa.hammad@zuj.edu.jo; https://orcid.org/0000-0003-3800-1220

**Alaa Khudair**: Faculty of Dentistry, Jordan University of Science and Technology, Amman, Jordan; alaakhudeir@gmail.com

**Short Title:** HLA-Class II Genes and Multiple Sclerosis in Jordanian Patients

**Supporting Information**

**Table S1.** Frequency of *HLA-DRB1* and *HLA-DQB1* allele among MS patients with optic neuritis and without optic neuritis

| **Allele** | **MS with optic neuritis** | | **MS without optic neuritis** | |  |  |  |  |
| --- | --- | --- | --- | --- | --- | --- | --- | --- |
| ***HLA-DRB1**** | **2N = 46** | **Allele Frequency (%)** | **2N = 84** | **Allele Frequency (%)** | ***P*** | ***Pc*** | **OR** | **95% CI** |
| *01:01* | 1 | 2.2 | 1 | 1.2 | 0.663 | - | 1.844 | 0.113-30.194 |
| *03:01* | 9 | 19.6 | 16 | 19.0 | 0.943 | - | 1.034 | 0.416-2.567 |
| *04:01* | 1 | 2.2 | 6 | 7.1 | 0.23 | - | 0.289 | 0.034-2.477 |
| *07:01* | 1 | 2.2 | 10 | 11.9 | 0.057 | - | 0.164 | 0.02-1.328 |
| *08:01* | 2 | 4.3 | 1 | 1.2 | 0.252 | - | 3.773 | 0.333-42.774 |
| *09:01* | 0 | 0 | 1 | 1.2 | 0.755 | - | 0.598 | 0.024-14.991 |
| *10:01* | 0 | 0 | 2 | 2.4 | 0.507 | - | 0.355 | 0.017-7.549 |
| *11:01* | 10 | 21.7 | 18 | 21.4 | 0.967 | - | 1.019 | 0.425-2.434 |
| *11:02* | 1 | 2.2 | 9 | 10.7 | 0.081 | - | 0.185 | 0.023-1.51 |
| *12:01* | 1 | 2.2 | 4 | 4.8 | 0.463 | - | 0.444 | 0.048-4.098 |
| *13:01* | 2 | 4.3 | 3 | 3.6 | 0.826 | - | 1.227 | 0.198-7.624 |
| *13:02* | 0 | 0 | 1 | 1.2 | 0.755 | - | 0.598 | 0.024-14.991 |
| *13:03* | 1 | 2.2 | 2 | 2.4 | 0.94 | - | 0.911 | 0.08-10.327 |
| *13:05* | 0 | 0 | 1 | 1.2 | 0.755 | - | 0.598 | 0.024-14.991 |
| *14:01* | 1 | 2.2 | 1 | 1.2 | 0.663 | - | 1.844 | 0.113-30.194 |
| ***15:01*** | **16** | **34.8** | **7** | **8.3** | **<0.001** | **<0.001** | **5.867** | **2.195-15.685** |
| *16:02* | 0 | 0 | 1 | 1.2 | 0.755 | - | 0.598 | 0.024-14.991 |
| ***HLA-DQB1**** |  |  |  |  |  | - |  |  |
| *02:01* | 11 | 23.9 | 25 | 29.8 | 0.476 | - | 0.742 | 0.326-1.69 |
| *03:01* | 11 | 23.9 | 22 | 26.2 | 0.775 | - | 0.886 | 0.385-2.039 |
| *03:02* | 6 | 13.0 | 5 | 6.0 | 0.165 | - | 2.37 | 0.682-8.241 |
| *03:03* | 0 | 0 | 3 | 3.6 | 0.363 | - | 0.25 | 0.013-4.955 |
| *04:01* | 0 | 0 | 3 | 3.6 | 0.363 | - | 0.25 | 0.013-4.955 |
| *05:01* | 4 | 8.7 | 9 | 10.7 | 0.092 | - | 0.4 | 0.13 - 1.19 |
| *05:02* | 0 | 0 | 1 | 1.2 | 0.755 | - | 0.598 | 0.024-14.991 |
| ***06:01*** | **5** | **10.9** | **2** | **2.4** | **0.04** | **0.012** | **5** | **0.93-26.885** |
| *06:02* | 9 | 19.6 | 14 | 16.7 | 0.679 | - | 1.216 | 0.481-3.074 |

N: number of volunteers, *Pc*: Corrected *P* value ≤ 0.013 OR: odds ratio, CI: Confidence Interval
