# Supplementary material for: HLA-class II genes association with multiple sclerosis: An immunogenetic prediction among multiple sclerosis Jordanian patients
Source: PLoS One. 2025 Feb 25;20(2):e0318824. doi: 10.1371/journal.pone.0318824 (PMC11856260; doi:10.1371/journal.pone.0318824)
Supplement: S2 Table — N: number of volunteers, Pc: Corrected P value ≤ 0.013 OR: odds ratio, CI: Confidence Interval. (DOCX) [file pone.0318824.s002.docx]

**HLA-Class II Genes Association with Multiple Sclerosis: An Immunogenetic Prediction Among Multiple Sclerosis Jordanian Patients**

Sawsan I. Khdair^1,*^, Lubna Al-Khareisha^1,2^, Osama H. Abusara^1^, Alaa M. Hammad^1^, Alaa Khudair^3^

^1^ Faculty of Pharmacy, Al-Zaytoonah University of Jordan, Amman 11733, Jordan

^2^ Department of Pharmacy, Al-Bashir Hospital, Amman, Jordan

^3^ Faculty of Dentistry, Jordan University of Science and Technology, Amman, Jordan

^*^ Corresponding author:

E-mail: sawsan.khdair@zuj.edu.jo (S.I. Khdair).

**Sawsan I. Khdair**: Faculty of Pharmacy, Al-Zaytoonah University of Jordan, Amman 11733, Jordan; [sawsan.khdair@zuj.edu.jo](mailto:sawsan.khdair@zuj.edu.jo); <https://orcid.org/0000-0002-1555-1062>

**Lubna Al-Khareisha**: Department of Pharmacy, Al-Bashir Hospital, Amman, Jordan; Faculty of Pharmacy, Al-Zaytoonah University of Jordan, Amman 11733, Jordan; alkhreishahlubna@gmail.com; https://orcid.org/0009-0001-6662-5949

**Osama H. Abusara**: Faculty of Pharmacy, Al-Zaytoonah University of Jordan, Amman 11733, Jordan; [o.abusara@zuj.edu.jo](mailto:o.abusara@zuj.edu.jo); <https://orcid.org/0000-0002-0856-5618>

**Alaa M. Hammad**: Faculty of Pharmacy, Al-Zaytoonah University of Jordan, Amman 11733, Jordan; alaa.hammad@zuj.edu.jo; https://orcid.org/0000-0003-3800-1220

**Alaa Khudair**: Faculty of Dentistry, Jordan University of Science and Technology, Amman, Jordan; alaakhudeir@gmail.com

**Short Title:** HLA-Class II Genes and Multiple Sclerosis in Jordanian Patients

**Supporting Information**

**Table S2.** The frequency of *HLA-DRB1~HLA-DQB1* haplotype among MS patients with and without optic neuritis in Jordanian population.

| **Haplotype** | **MS with optic neuritis** | | **MS without optic neuritis** | |  |  |  |  |
| --- | --- | --- | --- | --- | --- | --- | --- | --- |
| ***HLA-DRB1*~HLA-DQB1**** | **2N = 46** | **Allele Frequency (%)** | **2N = 84** | **Allele Frequency (%)** | ***P*** | ***Pc*** | **OR** | **95% CI** |
| *01:01~05:01* | 1 | 2.2 | 1 | 1.2 | 0.663 | - | 1.844 | 0.113-30.194 |
| *03:01~02:01* | 9 | 19.6 | 10 | 11.9 | 0.237 | - | 1.8 | 0.673-4.811 |
| *03:01~03:01* | 0 | 0 | 2 | 2.4 | 0.507 | - | 0.355 | 0.017-7.549 |
| *03:01~03:02* | 0 | 0 | 1 | 1.2 | 0.755 | - | 0.598 | 0.024-14.991 |
| *03:01~06:02* | 0 | 0 | 2 | 2.4 | 0.507 | - | 0.355 | 0.017-7.549 |
| *04:01~03:01* | 0 | 0 | 3 | 3.6 | 0.363 | - | 0.25 | 0.013-4.955 |
| *04:01~03:02* | 1 | 2.2 | 2 | 2.4 | 0.94 | - | 0.911 | 0.08-10.327 |
| *04:01~06:01* | 0 | 0 | 1 | 1.2 | 0.755 | - | 0.598 | 0.024-14.991 |
| *07:01~02:01* | 1 | 2.2 | 8 | 9.5 | 0.114 | - | 0.211 | 0.026-1.744 |
| *07:01~03:01* | 0 | 0 | 1 | 1.2 | 0.755 | - | 0.598 | 0.024-14.991 |
| *07:01~03:03* | 0 | 0 | 1 | 1.2 | 0.755 | - | 0.598 | 0.024-14.991 |
| *08:01~03:01* | 1 | 2.2 | 0 | 0 | 0.296 | - | 5.571 | 0.222-139.567 |
| *08:01~04:01* | 0 | 0 | 1 | 1.2 | 0.755 | - | 0.598 | 0.024-14.991 |
| *08:01~03:02* | 1 | 2.2 | 0 | 0 | 0.296 | - | 5.571 | 0.222-139.567 |
| *09:01~03:03* | 0 | 0 | 1 | 1.2 | 0.755 | - | 0.598 | 0.024-14.991 |
| *10:01~05:01* | 0 | 0 | 2 | 2.4 | 0.507 | - | 0.355 | 0.017-7.549 |
| *11:01~02:01* | 1 | 2.2 | 3 | 3.6 | 0.659 | - | 0.6 | 0.061-5.939 |
| *11:01~03:01* | 9 | 19.6 | 9 | 10.7 | 0.162 | - | 2.027 | 0.742-5.534 |
| *11:01~03:02* | 0 | 0 | 2 | 2.4 | 0.507 | - | 0.355 | 0.017-14.991 |
| *11:01~05:01* | 0 | 0 | 4 | 4.8 | 0.272 | - | 0.192 | 0.01-3.653 |
| *11:01~05:02* | 0 | 0 | 1 | 1.2 | 0.755 | - | 0.598 | 0.024-14.991 |
| *11:01~06:02* | 1 | 2.2 | 0 | 0 | 0.296 | - | 5.571 | 0.222-139.567 |
| *11:02~02:01* | 0 | 0 | 1 | 1.2 | 0.755 | - | 0.598 | 0.024-14.991 |
| *11:02~03:01* | 0 | 0 | 3 | 3.6 | 0.363 | - | 0.25 | 0.013-4.955 |
| *11:02~06:02* | 0 | 0 | 5 | 6.0 | 0.211 | - | 0.155 | 0.008-2.875 |
| *12:01~02:01* | 0 | 0 | 2 | 2.4 | 0.507 | - | 0.355 | 0.017-14.991 |
| *12:01~03:01* | 0 | 0 | 2 | 2.4 | 0.507 | - | 0.355 | 0.017-14.991 |
| *12:01~03:02* | 1 | 2.2 | 0 | 0 | 0.296 | - | 5.571 | 0.222-139.567 |
| *13:01~04:01* | 0 | 0 | 1 | 1.2 | 0.755 | - | 0.598 | 0.024-14.991 |
| *13:01~05:01* | 0 | 0 | 1 | 1.2 | 0.755 | - | 0.598 | 0.024-14.991 |
| *13:01~06:02* | 2 | 4.3 | 1 | 1.2 | 0.252 | - | 3.773 | 0.333-42.774 |
| *13:02~05:01* | 0 | 0 | 1 | 1.2 | 0.755 | - | 0.598 | 0.024-14.991 |
| *13:03~03:01* | 1 | 2.2 | 2 | 2.4 | 0.94 | - | 0.911 | 0.08-10.327 |
| *13:05~03:01* | 0 | 0 | 1 | 1.2 | 0.755 | - | 0.598 | 0.024-14.991 |
| *14:01~05:01* | 1 | 2.2 | 0 | 0 | 0.296 | - | 5.571 | 0.222-139.567 |
| *14:01~06:02* | 0 | 0 | 1 | 1.2 | 0.755 | - | 0.598 | 0.024-14.991 |
| *15:01~03:02* | 4 | 8.7 | 0 | 0 | 0.055 | - | 17.894 | 0.9412-340.163 |
| *15:01~05:01* | 2 | 4.3 | 0 | 0 | 0.149 | - | 9.494 | 0.446-202.096 |
| *15:01~06:01* | 4 | 8.7 | 1 | 1.2 | **0.033** | 0.033 | 7.905 | 0.856-72.965 |
| *15:01~06:02* | 6 | 13.0 | 6 | 7.1 | 0.266 | - | 1.95 | 0.591-6.436 |
| *16:02~03:01* | 0 | 0 | 1 | 1.2 | 0.755 | - | 0.598 | 0.024-14.991 |

N: number of volunteers, *Pc*: Corrected *P* value ≤ 0.013 OR: odds ratio, CI: Confidence Interval
