# Supplementary material for: HLA-class II genes association with multiple sclerosis: An immunogenetic prediction among multiple sclerosis Jordanian patients
Source: PLoS One. 2025 Feb 25;20(2):e0318824. doi: 10.1371/journal.pone.0318824 (PMC11856260; doi:10.1371/journal.pone.0318824)
Supplement: S3 Table — N: number of volunteers, Pc: Corrected P value ≤ 0.013 OR: odds ratio, CI: Confidence Interval. (DOCX) [file pone.0318824.s003.docx]

**HLA-Class II Genes Association with Multiple Sclerosis: An Immunogenetic Prediction Among Multiple Sclerosis Jordanian Patients**

Sawsan I. Khdair^1,*^, Lubna Al-Khareisha^1,2^, Osama H. Abusara^1^, Alaa M. Hammad^1^, Alaa Khudair^3^

^1^ Faculty of Pharmacy, Al-Zaytoonah University of Jordan, Amman 11733, Jordan

^2^ Department of Pharmacy, Al-Bashir Hospital, Amman, Jordan

^3^ Faculty of Dentistry, Jordan University of Science and Technology, Amman, Jordan

^*^ Corresponding author:

E-mail: sawsan.khdair@zuj.edu.jo (S.I. Khdair).

**Sawsan I. Khdair**: Faculty of Pharmacy, Al-Zaytoonah University of Jordan, Amman 11733, Jordan; [sawsan.khdair@zuj.edu.jo](mailto:sawsan.khdair@zuj.edu.jo); <https://orcid.org/0000-0002-1555-1062>

**Lubna Al-Khareisha**: Department of Pharmacy, Al-Bashir Hospital, Amman, Jordan; Faculty of Pharmacy, Al-Zaytoonah University of Jordan, Amman 11733, Jordan; alkhreishahlubna@gmail.com; https://orcid.org/0009-0001-6662-5949

**Osama H. Abusara**: Faculty of Pharmacy, Al-Zaytoonah University of Jordan, Amman 11733, Jordan; [o.abusara@zuj.edu.jo](mailto:o.abusara@zuj.edu.jo); <https://orcid.org/0000-0002-0856-5618>

**Alaa M. Hammad**: Faculty of Pharmacy, Al-Zaytoonah University of Jordan, Amman 11733, Jordan; alaa.hammad@zuj.edu.jo; https://orcid.org/0000-0003-3800-1220

**Alaa Khudair**: Faculty of Dentistry, Jordan University of Science and Technology, Amman, Jordan; alaakhudeir@gmail.com

**Short Title:** HLA-Class II Genes and Multiple Sclerosis in Jordanian Patients

**Supporting Information**

**Table S3.** The frequency of *HLA-DRB1* and *HLA-DQB1* alleles among MS patients with sensory impairment and without sensory impairment.

| **Allele** | **MS with sensory impairment** | | **MS without sensory impairment** | |  |  |  |  |
| --- | --- | --- | --- | --- | --- | --- | --- | --- |
| ***HLA-DRB1**** | **2N = 56** | **Allele Frequency (%)** | **2N = 74** | **Allele Frequency (%)** | ***P*** | ***Pc*** | **OR** | **95% CI** |
| *01:01* | 2 | 3.6 | 0 | 0 | 0.218 | - | 6.835 | 0.322-145.255 |
| *03:01* | 13 | 23.2 | 12 | 16.2 | 0.316 | - | 1.562 | 0.651-3.75 |
| *04:01* | 1 | 1.8 | 6 | 8.1 | 0.114 | - | 0.206 | 0.024-1.763 |
| *07:01* | 7 | 12.5 | 4 | 5.4 | 0.15 | - | 2.5 | 0.694-9.006 |
| *08:01* | 3 | 5.4 | 0 | 0 | 0.135 | - | 9.748 | 0.493-192.677 |
| *09:01* | 0 | 0 | 1 | 1.4 | 0.611 | - | 0.434 | 0.017-10.847 |
| *10:01* | 2 | 3.6 | 0 | 0 | 0.218 | - | 6.835 | 0.322-145.255 |
| *11:01* | 10 | 17.9 | 18 | 24.3 | 0.374 | - | 0.676 | 0.285-1.608 |
| *11:02* | 1 | 1.8 | 9 | 12.2 | **0.028** | 0.027 | 0.131 | 0.016-1.069 |
| *12:01* | 1 | 1.8 | 4 | 5.4 | 0.288 | - | 0.318 | 0.035-2.928 |
| *13:01* | 1 | 1.8 | 4 | 5.4 | 0.288 | - | 0.318 | 0.035-2.928 |
| *13:03* | 2 | 3.6 | 1 | 1.4 | 0.404 | - | 2.704 | 0.239-30.591 |
| *13:05* | 0 | 0 | 1 | 1.4 | 0.611 | - | 0.434 | 0.017-10.847 |
| *14:01* | 1 | 1.8 | 1 | 1.4 | 0.842 | - | 1.327 | 0.081-21.692 |
| *15:01* | 11 | 19.6 | 12 | 16.2 | 0.612 | - | 1.263 | 0.512-3.118 |
| *16:02* | 1 | 1.8 | 1 | 1.4 | 0.842 | - | 1.327 | 0.081-21.692 |
| ***HLA-DQB1**** |  |  |  |  |  |  |  |  |
| *02:01* | 18 | 32.1 | 18 | 24.3 | 0.324 | - | 1.474 | 0.681-3.19 |
| *03:01* | 14 | 25.2 | 19 | 25.7 | 0.93 | - | 0.965 | 0.434-2.145 |
| *03:02* | 5 | 8.9 | 6 | 8.1 | 0.868 | - | 1.111 | 0.321-3.844 |
| *03:03* | 0 | 0 | 3 | 4.1 | 0.261 | - | 0.181 | 0.009-3.573 |
| *04:01* | 1 | 1.8 | 2 | 2.7 | 0.73 | - | 0.655 | 0.095-2.737 |
| ***05:01*** | **12** | **21.4** | **1** | **1.4** | **<0.001** | **<0.001** | **19.909** | **2.502-158.41** |
| *05:02* | 0 | 0 | 1 | 1.4 | 0.611 | - | 0.434 | 0.017-10.847 |
| *06:01* | 2 | 3.6 | 5 | 6.8 | 0.426 | - | 0.511 | 0.434-2.145 |
| ***06:02*** | **4** | **7.4** | **19** | **25.7** | **0.006** | **0.006** | **0.223** | **0.071-0.698** |

N: number of volunteers, *Pc*: Corrected *P* value ≤ 0.013 OR: odds ratio, CI: Confidence Interval
