# Supplementary material for: HLA-class II genes association with multiple sclerosis: An immunogenetic prediction among multiple sclerosis Jordanian patients
Source: PLoS One. 2025 Feb 25;20(2):e0318824. doi: 10.1371/journal.pone.0318824 (PMC11856260; doi:10.1371/journal.pone.0318824)
Supplement: S4 Table — N: number of volunteers, Pc: Corrected P value ≤ 0.013 OR: odds ratio, CI: Confidence Interval. (DOCX) [file pone.0318824.s004.docx]

**HLA-Class II Genes Association with Multiple Sclerosis: An Immunogenetic Prediction Among Multiple Sclerosis Jordanian Patients**

Sawsan I. Khdair^1,*^, Lubna Al-Khareisha^1,2^, Osama H. Abusara^1^, Alaa M. Hammad^1^, Alaa Khudair^3^

^1^ Faculty of Pharmacy, Al-Zaytoonah University of Jordan, Amman 11733, Jordan

^2^ Department of Pharmacy, Al-Bashir Hospital, Amman, Jordan

^3^ Faculty of Dentistry, Jordan University of Science and Technology, Amman, Jordan

^*^ Corresponding author:

E-mail: sawsan.khdair@zuj.edu.jo (S.I. Khdair).

**Sawsan I. Khdair**: Faculty of Pharmacy, Al-Zaytoonah University of Jordan, Amman 11733, Jordan; [sawsan.khdair@zuj.edu.jo](mailto:sawsan.khdair@zuj.edu.jo); <https://orcid.org/0000-0002-1555-1062>

**Lubna Al-Khareisha**: Department of Pharmacy, Al-Bashir Hospital, Amman, Jordan; Faculty of Pharmacy, Al-Zaytoonah University of Jordan, Amman 11733, Jordan; alkhreishahlubna@gmail.com; https://orcid.org/0009-0001-6662-5949

**Osama H. Abusara**: Faculty of Pharmacy, Al-Zaytoonah University of Jordan, Amman 11733, Jordan; [o.abusara@zuj.edu.jo](mailto:o.abusara@zuj.edu.jo); <https://orcid.org/0000-0002-0856-5618>

**Alaa M. Hammad**: Faculty of Pharmacy, Al-Zaytoonah University of Jordan, Amman 11733, Jordan; alaa.hammad@zuj.edu.jo; https://orcid.org/0000-0003-3800-1220

**Alaa Khudair**: Faculty of Dentistry, Jordan University of Science and Technology, Amman, Jordan; alaakhudeir@gmail.com

**Short Title:** HLA-Class II Genes and Multiple Sclerosis in Jordanian Patients

**Supporting Information**

**Table S4.** Frequency of *HLA-DRB1* alleles among MS patients with brainstem symptoms and without brainstem symptoms

N: number of volunteers, *Pc*: Corrected *P* value ≤ 0.013 OR: odds ratio, CI: Confidence Interval

| **Allele** | **MS with brainstem symptom** | | **MS without brainstem symptom** | |  |  |  |  |
| --- | --- | --- | --- | --- | --- | --- | --- | --- |
| ***HLA-DRB1**** | **2N=20** | **(%)** | **2N=45** | **(%)** | ***P*** | ***Pc*** | **OR** | **95% CI** |
| **01:01* | 0 | 0 | 2 | 2.2 | 0.596 | - | 0.437 | 0.021-9.313 |
| **03:01* | 7 | 17.5 | 18 | 20.0 | 0.739 | - | 0.848 | 0.323-2.228 |
| **04:01* | 2 | 5.0 | 5 | 5.6 | 0.897 | - | 0.895 | 0.166-4.819 |
| ****07:01*** | **7** | **17.5** | **4** | **4.4** | **0.001** | **0.001** | **4.561** | **1.252-16.607** |
| **08:01* | 2 | 5.0 | 1 | 1.1 | 0.173 | - | 4.684 | 0.412-53.224 |
| **09:01* | 0 | 0 | 1 | 1.1 | 0.853 | - | 0.737 | 0.029-18.475 |
| **10:01* | 0 | 0 | 2 | 2.2 | 0.596 | - | 0.437 | 0.021-9.313 |
| **11:01* | 9 | 22.5 | 19 | 21.1 | 0.859 | - | 1.085 | 0.442-2.664 |
| **11:02* | 4 | 10 | 6 | 6.7 | 0.51 | - | 1.556 | 0.414-5.847 |
| **12:01* | 1 | 2.5 | 4 | 4.4 | 0.595 | - | 0.551 | 0.06-5.095 |
| **13:01* | 0 | 0 | 5 | 5.6 | 0.268 | - | 0.192 | 0.01-3.555 |
| **13:02* | 0 | 0 | 1 | 1.1 | 0.853 | - | 0.737 | 0.029-18.475 |
| **13:03* | 0 | 0 | 3 | 3.3 | 0.44 | - | 0.309 | 0.016-6.117 |
| **13:05* | 0 | 0 | 1 | 1.1 | 0.853 | - | 0.737 | 0.029-18.475 |
| **14:01* | 0 | 0 | 2 | 2.2 | 0.596 | - | 0.437 | 0.021-9.313 |
| **15:01* | 8 | 20.0 | 15 | 16.7 | 0.646 | - | 1.25 | 0.482-3.241 |
| **16:02* | 0 | 0 | 1 | 1.1 | 0.853 | - | 0.737 | 0.029-18.475 |
