# Supplementary material for: HLA-class II genes association with multiple sclerosis: An immunogenetic prediction among multiple sclerosis Jordanian patients
Source: PLoS One. 2025 Feb 25;20(2):e0318824. doi: 10.1371/journal.pone.0318824 (PMC11856260; doi:10.1371/journal.pone.0318824)
Supplement: Attachment — Submitted filename: Manuscript Review.docx [file pone.0318824.s005.docx]

**Manuscript Review**

**Manuscript ID:** PONE-D-24-50217

**Title:** HLA-Class II Genes Association with Multiple Sclerosis: An Immunogenetic Prediction Among Multiple Sclerosis Jordanian Patients

**Journal**: Journal of Immunoassay and Immunochemistry

**Summary and general comments:**

In the manuscript “**HLA-Class II Genes Association with Multiple Sclerosis: An Immunogenetic Prediction Among Multiple Sclerosis Jordanian Patients**” the authors took a correlation approach to assess the association between HLA class II genes and Multiple Sclerosis (MS). To achieve such aim, HLA class II genotyping by PCR SSP was performed in 65 MS patients comparatively to 75 healthy controls.

The main results showed:

1. Significant association of HLA-DRB1*03:01 and DRB1*0401 alleles with MS susceptibility
2. Significant association of HLA-DRB1*15:01 and DQB1*06:01 alleles with optic neuritis

Overall, the paper is well organized and I really enjoyed reading it. I believe it fits for publication in its current form.
